# Establishment of macrocyclic lactone resistant *Dirofilaria immitis* isolates in experimentally infected laboratory dogs

**DOI:** 10.1186/s13071-014-0494-6

**Published:** 2014-11-07

**Authors:** Cassan N Pulaski, John B Malone, Catherine Bourguinat, Roger Prichard, Timothy Geary, Danielle Ward, Thomas R Klei, Tal Guidry, George ‘Bud’ Smith, Brooke Delcambre, Jonathan Bova, Jenny Pepping, James Carmichael, Rudolf Schenker, Romain Pariaut

**Affiliations:** School of Veterinary Medicine, Louisiana State University and Louisiana Animal Disease Diagnostic Laboratory, Baton Rouge, 70803 LA USA; Institute of Parasitology, McGill University, 21 111 Lakeshore Road, Sainte-Anne de Bellevue, H9X3V9 Quebec Canada; Novartis Animal Health US, Inc, Greensboro, NC USA; Novartis Animal Health, Basel, Switzerland; Guidry Animal Hospital, 500 East Gloria Switch Rd, 70507 Lafayette, LA USA; Bocage Animal Hospital, 7353 Jefferson Hwy, 70806 Baton Rouge, LA USA

**Keywords:** *Dirofilaria immitis*, Canine heartworms, Macrocyclic lactone, Drug resistance, Lack of efficacy

## Abstract

**Background:**

Strains of *Dirofilaria immitis* suspected of lack of efficacy (LOE) to macrocyclic lactone (ML) preventive drugs have been increasingly reported in dogs by practicing veterinarians since 2005 in the Lower Mississippi Delta region. If proven, and not controlled in the early stages, the emergence of ML drug resistance threatens to become a widespread problem in the US that may limit the effectiveness of current preventive drug treatment methods.

**Methods:**

To validate practice reports, a statewide survey of Louisiana veterinarians was done to define the extent of the problem and identify focal ‘hotspots’ of reported ML LOEs using Geographic Information Systems (GIS) methods. The present study then utilized microfilariae (Mf) from two canine field cases from different state locations that fit criteria for a high index of suspicion of LOE against heartworms by ML drugs. Blood containing Mf from the canine field cases was used to infect and produce L_3_ in *Aedes aegypti* for experimental infection of two groups of dogs, each of which contained two laboratory dogs, one treated with prophylactic ivermectin (12 μg/kg) monthly for 6 months at twice the label dose (6 μg/kg), and one untreated control.

**Results:**

Both treated and untreated dogs from Group I and Group II developed patent *D. immitis* infections by 218 DPI and 189 DPI, respectively, as evidenced by a positive occult heartworm antigen test and microfilaremia by the Knott’s test. Mf counts gradually increased post-patency in test and control dogs. Infective larvae raised from microfilariae from the treated Group I dog were used to successfully establish a second generation isolate, confirming heritability of resistance in the face of a monthly ivermectin challenge dose of 24 μg/kg, given monthly for 3 months.

**Conclusions:**

These experimental infection studies provide *in vivo* evidence of the existence of ML drug resistance in dogs infected by *D. immitis* L_3_ from suspect field LOE cases in the Lower Mississippi Delta. Results encourage further work on mechanisms underlying the emergence of ML resistance in *D. immitis* and development of evidence-based resistance management strategies for heartworm preventives in order to extend the useful life of current drugs.

## Background

In 2005, an evaluation of the efficacy of heartworm preventive products was published by the Center for Veterinary Medicine, US Food and Drug Administration (FDA) that reported concerns of an increased number of reports by practicing veterinarians on lack of efficacy (LOE) of macrocyclic lactone (ML) preventive drugs [1]. Eighteen years after the first monthly ML drug formulation was approved in 1987, monthly ML drugs had largely replaced daily diethylcarbamazine, an approved treatment since 1977, to become the dominant means of preventing heartworms in companion animals in the USA. The first ML LOE reports to the FDA, beginning in 1998, had grown in number to several hundred in 1999–2000, nearly 1000 in 2000–2001 and over 1500 in 2002–2003 [1]. It was proposed that either true emerging resistance, enhanced FDA surveillance records systems or climate-based shifts in mosquito vector population species may have had a role.

In response to this, and anecdotal reports of increasing LOE cases from the veterinary community in heartworm endemic areas, pharmaceutical firms initiated reimbursement programs in 2004 to cover costs of treatment of LOE cases if owners and their veterinarians could document full compliance with recommended preventive treatment practices. Concurrently, several research efforts were initiated, investigating the spatio-temporal scale and dynamics of LOE reports [2], potential *in vitro* indicators of resistance [3,4], possible genetic markers of resistance via genomic studies [5-10] and *in vivo* experiments, the gold standard, to establish confirmed resistant strains from canine field cases in the laboratory [11,12].

The objectives of studies reported here are to record results of: 1) a 2009 survey to document the experience of veterinarians in Louisiana on potential emerging ML resistance by *D. immitis*, and 2) establishment of ML resistant isolates of *D. immitis* in experimentally infected laboratory dogs using L_3_ raised in *Aedes aegypti* fed on microfilaremic blood from 2 dogs with a ‘high index of suspicion of resistance’.

## Methods

### Objective 1: statewide practitioner survey

In August 2009, a one-page ‘check-off’ questionnaire survey was sent to all Louisiana veterinarians listed in the Louisiana Veterinary Medical Association (LVMA) database and in the 2008 billing records of the Louisiana Animal Disease Diagnostic Laboratory (LADDL). Fifteen survey questions queried whether ML LOE cases had been seen in client dogs, including year of the first ML LOE case, specific drug used on suspected LOE cases, and the number of ML LOE cases reported. A total of 855 surveys were sent.

Survey data results were entered into a Microsoft® excel (Microsoft Office, 2007) database and linked to a map of Louisiana within a geographic information system (GIS) according to the longitude/latitude geographic point location and Zip Code of each responding veterinarian’s clinic using ArcGIS 9.3 software (ESRI, Redlands, CA) [2].

### Objective 2: experimental infection studies

#### Field isolates

Microfilariae (Mf) positive blood from canine field cases from different state locations was collected from client-owned dogs for use in the current study. After review and approval of experimental protocols by the Louisiana State University Institutional Animal Care and Use Committee, owner consent forms informing clients of the study purpose and design were agreed upon and signed prior to enrollment in the study. Based on practitioner and client cooperation, two dogs were selected based on the following criteria for a ‘high index of suspicion of ML resistance’ by *D. immitis*:

- History of failure of efficacy and full monetary compensation by a commercial pharmaceutical firm;
- Residence in an area identified as a ‘hotspot’ of suspected ML drug resistance in the 2009 statewide veterinary practitioner survey of LOE cases [2];
- Persistence of circulating Mf following an accepted microfilaricidal dose of ML [8] and;
- High frequency of a genotype marker previously reported to be correlated with potential ML resistance, single nucleotide polymorphism at sites 11 and 618 (GG-GG) of a gene encoding for P-glycoprotein [5].

### Dogs

Four male hound-cross dogs of 4–5 months-of-age were purchased from a USDA approved commercial supplier for experimental infections. All dogs were housed strictly in indoor runs in a mosquito-free kennel facility confirmed by periodic overnight sampling using a CDC Miniature Light Trap, Model 512 (John W Hock Co., Gainesville, FL). All dogs tested negative for adult heartworm antigen (DiroCHEK® Canine Heartworm Antigen Test Kit, Symbiotics Corporation, San Diego, CA, USA) in serum samples collected prior to experimental infection and at 1, 2, 3, and 4 months post-infection. None of the dogs were treated with any ML in the holding period before experimental infection of dogs with *D. immitis* L_3_ at 12–15 months-of-age.

### Experimental design

Dogs were randomly assigned to two groups of two dogs for experimental infection by the two field isolates (Group I – “LSU 10” strain; Group II – “LSU 13” strain). At 27–33 days after infection, one dog in each group was treated with 12 μg/kg ivermectin diluted 1:9 in propylene glycol by the subcutaneous (SC) route. The untreated control dog received a similar volume of only propylene glycol by the SC route. At monthly intervals thereafter, dogs received respective ivermectin (12 μg/kg) or propylene glycol sham treatment for a total of 6 monthly treatments. Although not required to confirm resistance, the ivermectin monthly treatment was repeated six times, at double the recommended dose, in order to ensure the presence of resistance. Ivermectin drug (Merial, Ivomec® 1% Injection, 50 mL) was from batch number BA132/10 (certificate of analysis available).

### D. immitis infection

Blood containing Mf from the two canine field cases was collected and used to infect laboratory-raised *Aedes aegypti* mosquitos using a membrane feeding apparatus (Thermo NESLAB, Model RTE-111, Neslab Instruments, Newington, NH, USA). Mosquitoes were infected and held in an insectary for 14–16 days (27°C, 80% RH, 12 hour light cycle) prior to harvesting L_3_ according to the procedure of the NIAID/NIH Filariasis Research Reagent Repository Center (FR3), Athens, GA, USA [13]. Each dog received a SC inoculation in the inguinal area containing infective third-stage *D immitis*. Recovered L_3_ were held in RPMI tissue culture media at room temperature for up to 3 hours until SC inoculation. Group I (LSU 10) dogs each received 69 L_3_ (31 on 23rd March 2012 and 38 on 30th March 2012) and Group II (LSU 13) dogs each received 75 L_3_ (test) or 114 L_3_ (untreated control) on 15th June 2012. Group I dogs were infected twice, one week apart, because an adequate number of L_3_ larvae were not obtained following the initial harvest.

Whole blood samples were collected from each dog at monthly intervals for four months following experimental infection_,_ and at two week intervals thereafter, to determine *D. immitis* infection status and the onset of patency. Blood samples were analyzed for adult heartworm antigen (Dirochek® Canine Heartworm Antigen Test Kit, Symbiotics Corporation, San Diego, CA) and the presence of microfilariae was monitored using a 20 μL direct blood smear and the modified Knott’s technique [14]. Results were recorded over time as number of days post-infection (DPI).

### Heritability

In separate follow-up studies, infective larvae raised from microfilariae from the treated Group I dog (LSU 10 strain) were used to confirm heritability of resistance in the face of a monthly ivermectin challenge dose of 24 μg/kg for 3 monthly treatments. Similar procedures to those previously described were used to infect (45 L_3_) and monitor the dog used to establish this second generation isolate of the LSU 10 strain (LSU 10-II). An ivermectin treatment dose at 4 times the recommended preventive level was used to further confirm strain resistance, which had previously been assessed at twice the recommended level.

### Ethical approval

This study was reviewed and approved by the Louisiana State University Institutional Animal Care and Use Committee (PRN 13–072).

## Results

### Statewide survey

**Figure 1:**
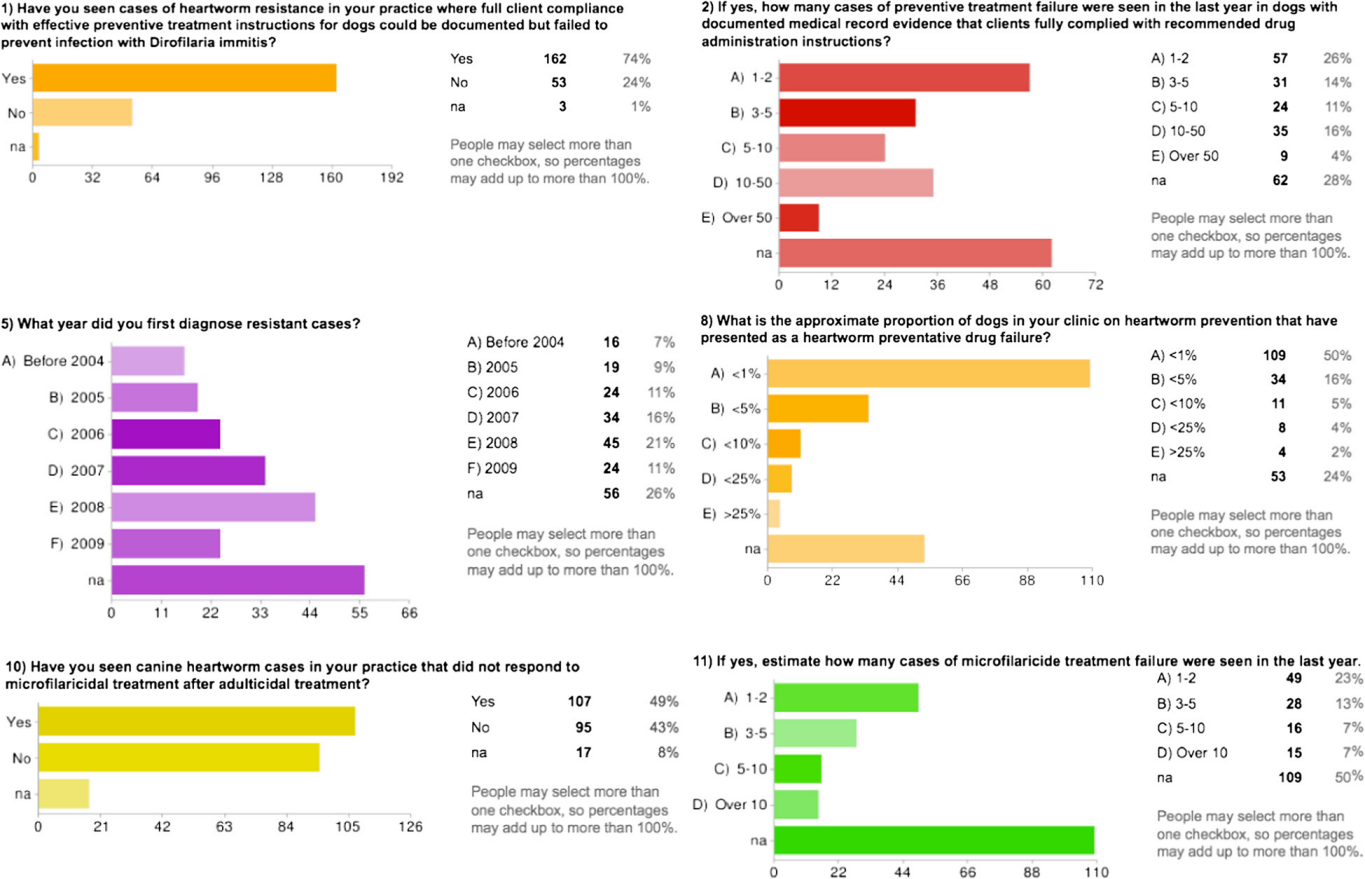
**Pertinent survey questions with results.**

Of the 855 one-page mail surveys sent to all Louisiana practitioners in August 2009 regarding their opinions of ML LOEs reported within their clinics, 221 were returned (25.8% response rate). Of the 221 surveys submitted, 70% were from clinics classified as ‘small animal’ and 25% were from ‘mixed animal’ facilities. GIS analyses of survey results indicated there were focal locations with veterinarian perceived high rates of reported LOEs of ML drugs against heartworms and that numbers increased from 2005 to 2008. Pertinent survey questions with results are shown in Figure 1 [2].

### Experimental infections

Both treated and untreated dogs from Group I (LSU 10) and Group II (LSU 13) developed patent *D. immitis* infections by 218 DPI and 189 DPI, respectively, as evidenced by a positive occult heartworm antigen test and microfilaremia by the modified Knott’s test. Mf counts gradually increased post-patency in test and control dogs (Table 1).

**Table 1:** **Monthly parasitological monitoring**

**Name**	**DOI**	**Date**	**26Oct12**	**27Nov12**	**20Dec12**	**18Jan13**	**18Feb13**	**15Mar13**	**19Apr13**	**17May13**	**14Jun13**	**12Jul13**	**14Aug13**	**13Sep13**	**7Nov13**	**9Dec13**	**10Jan14**
LSU10a	23-Mar-12	Occult	***+***	+	+	+	+	+	+	+	+	+	+	+	+	+	+
		DS	***0***	3	7	1	12	8	21	12	10	14	1	ND	6	7	ND
		Knotts	***7***	36	345	516	869	684	130	1438	615	1342	8	ND	ND	ND	2
LSU10b	23-Mar-12	Occult	***+***	+	+	+	+	+	+	+	+	+	+	+	+	+	+
		DS	***6***	9	100	26	74	33	443	ND	631	640	621	ND	317	79	ND
		Knotts	***526***	848	2610	196	374	1723	9460	9020	8294	16082	ND	ND	ND	ND	ND
		**DPI**	**218**	**250**	**273**	**302**	**333**	**358**	**393**	**421**	**449**	**477**	**510**	**540**	**595**	**627**	**659**
LSU13a	15-Jun-12	Occult	-	+	***+***	+	+	+	+	+	+	+	+	+	+	+	+
		DS	0	0	***0***	1	27	22	307	ND	543	562	767	ND	315	145	ND
		Knotts	0	0	***2***	104	1222	1723	6878	7810	8767	12254	ND	ND	ND	ND	ND
LSU13b	15-Jun-12	Occult	-	+	***+***	+	+	+	+	+	+	+	+	+	+	+	+
		DS	0	0	***0***	1	52	26	263	ND	817	89	1047	ND	536	300	ND
		Knotts	0	0	***5***	76	3480	1672	5222	11770	19019	21516	3256	ND	ND	ND	ND
		**DPI**	**134**	**166**	**189**	**218**	**249**	**274**	**309**	**337**	**365**	**393**	**426**	**456**	**511**	**543**	**575**
LSU10-II	22-Feb-13	Occult						-	-	-	-	-	-	+	*+*	+	+
		DS						0	0	0	0	0	0	ND	***5***	13	ND
		Knotts						0	0	0	0	0	0	ND	***ND***	ND	1019
		**DPI**	**N/A**	**N/A**	**N/A**	**N/A**	**N/A**	**22**	**57**	**85**	**113**	**141**	**174**	**204**	**259**	**291**	**323**

Bold, italicized values indicate first detection of circulating Mf DOI, Date of Infection; Occult, Adult Heartworm Antigen Test; DS, Direct Smear using 20 μL of whole blood; Knotts, Modified Knott’s Technique; DPI, Days Post-Infection; ND, No Data.

### Heritability of resistance

Microfilaremia in one of the dogs (LSU 10a), initially found in moderate numbers, became sporadic with biweekly counts of 2–8 Mf per ml of blood. Microfilariae from this dog (LSU 10a) were used to 1) maintain an adequate Mf level of LSU 10 strain to infect mosquitos for second generation passage of the to a new laboratory dog (LSU 10-II); 2) to confirm resistance in the face of challenge treatment by a 24 μg/kg dose of ivermectin given monthly for 3 months, and 3) to demonstrate heritability of the LSU 10 resistant strain. Monthly monitoring of the LSU 10-II dog showed it became positive for circulating antigen by 204 DPI and became patent with circulating Mf by 259 DPI.

Five laboratory dogs harboring resistant strain *D. immitis* isolates from two separate client-owned dogs (LSU 10, LSU 13) are thus currently available for further study:

### Dog challenge treatment

LSU 10a ivermectin (12 μg/kg, monthly for 6 months)

LSU 10b untreated control

LSU 10-II ivermectin (24 μg/kg, monthly for 3 months)

LSU 13a ivermectin (12 μg/kg, monthly for 6 months)

LSU 13b untreated control

We propose that we have successfully isolated two strains from proven cases of resistance in client-owned dogs in Louisiana that can be used to further study and characterize the emergence of ML resistance by *D. immitis.*

Genomics studies

Samples of 20–30 individual Mf, from Group I and Group II *D. immitis* infected dogs, were collected, preserved in isopropyl alcohol, and analyzed blindly in order to genotype the circulating Mf population for two single nucleotide polymorphism loci on a P-glycoprotein gene previously shown to be correlated with a loss of efficacy of macrocyclic lactone heartworm anthelminthics [5-7]. The investigation of percentage frequencies of GG-GG genotype in the different groups was the main interest. DNA extraction of individual Mf had been extracted following the protocol of QIAamp DNA Micro kit from Qiagen® (www.qiagen.com). Also, individual Mf DNA samples had been amplified using Repli-g screening kit from Qiagen®, to increase DNA concentration of individual samples. Individual genotypes had been identified after PCR, Sanger sequencing, and analysis of chromatograms using Sequencher® (http://genecodes.com/). Table 2 shows results of preliminary studies on Mf from the original two client owned dogs (LSU 10, LSU 13) used to establish resistant strains in the laboratory as compared to Mf from two dogs from a police dog kennel (LSU 11, LSU 14). Results showed a higher percentage frequency of GG-GG genotype in Mf populations from LSU 10 and LSU 13 strain dogs compared to Mf population from LSU 11 and LSU 14.

**Table 2:** **Genomics studies**

	**Percentage frequency %**										
	**AA-AA**	**AA-GG**	**AA-AG**	**AG-GG**	**GG-GG**	**GG-AA**	**??-GG**	**??-AA**	**??-AG**	**Total**	**N**
**LSU10**	10.0	13.3	3.3	0.0	**40.0**	3.3	23.3	3.3	3.3	100.0	30
**LSU11**	11.1	70.4	3.7	0.0	7.4	0.0	7.4	0.0	0.0	100.0	27
**LSU13**	0.0	41.4	0.0	6.9	**51.7**	0.0	0.0	0.0	0.0	100.0	29
**LSU14**	8.7	60.9	4.4	0.0	17.4	0.0	8.7	0.0	0.0	100.0	23

Preliminary study of PCR analysis of 23–30 individual microfilariae showing high frequency of the GG-GG marker from dogs harboring proven resistant strains (indicated in bold) as compared to pooled microfilariae from two infected dogs from a local police dog kennel. ??, Results Inconclusive (position 11 undetermined, position 618 determined).

## Discussion

The aim of the current study was to provide evidence to test the following hypothesis – ‘Resistant strain(s) of *D. immitis* exist in the general population of Louisiana dogs, and these resistant strains can be identified by case history, *in vivo* tests and genetic markers to enable development of effective alternative drug therapies to extend the useful life of current preventive drugs’.

**Figure 2:**
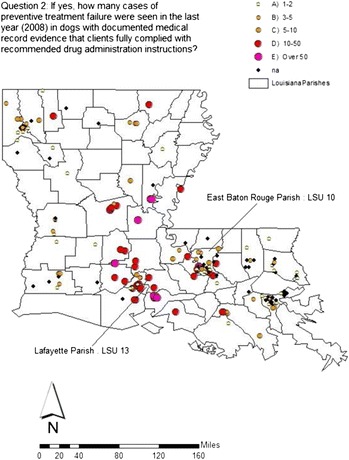
**Survey question #2 with field isolate residences noted.**

Beginning in 2005, LOE reimbursement programs by pharmaceutical firms provided an opportunity to study pre-screened records of possible resistance and made it more likely to identify sporadic cases of resistance, if present, in the general population of dogs in South Louisiana. The ‘microfilariae reduction test’ proposed by Geary *et al.* [8] provided a simple method for testing potential drug failure that could be used in clinical settings when resistance was suspected. Initial studies on the ‘Katrina dog’ [5] and other suspect cases [6,7] provided a potential new tool to find cases based on genetic markers of ML resistance by *D immitis*. Finally, a statewide survey was done of Louisiana veterinary practices in 2009 to identify the extent and geospatial distribution of company reimbursed cases (Figures 1 and 2) [2].

Statewide survey results indicated that current drugs were still effective in most practices, but that troubling hot spots of suspected resistance were emerging. Some practices in high prevalence areas had over 50 cases per year, some reported resistance in up to 25% of dogs under their care, and 74% of practices indicated they had seen at least one LOE case in the past year. Faced with the uncertainty presented by apparent frequent drug failure, especially in large breed outside dogs subjected to bites from large mosquito populations, practices in some areas abandoned the commonly used monthly preventive drugs and began searching for alternative preventive protocols, including use of monthly preventive drugs administered at higher doses, long-acting injectable or topical ML drugs that maintain high continuous blood levels [8], or strategic use of doxycycline in combination with ML drugs [15-17]. Some of the long-acting ML drugs have additional label claims of efficacy against other helminthes in which ML is given at elevated doses above that needed for HW prevention alone [9]. Controversies continued, as explanations, including environmental changes, vector population shifts, and owner compliance issues, for the growing number of LOE cases multiplied. There was a clear need to establish the ‘gold standard’ of experimental isolation and characterization of suspect cases of canine heartworm resistance in controlled experiments.

We adopted a four point ‘high index of suspicion’ criteria to enable identification and isolation of resistant strains of *D. immitis:* a) clinical history of failure of efficacy and full monetary compensation by a commercial pharmaceutical firm; b) residence in an area identified as a ‘hotspot’ of suspected drug resistance in the 2009 statewide veterinary practitioner survey of LOE cases [2]; c) persistence of circulating Mf seven days after an accepted microfilaricidal dose of ML [8] and d) high frequency of a genotype marker previously reported to be correlated with potential ML resistance, single nucleotide polymorphism at sites 11 and 618 (GG-GG) of a gene encoding for P-glycoprotein [5].

Using these criteria for high index of suspicion, LADDL began searching for cases in high prevalence foci of infection in Louisiana by retrospective study of positive confirmatory antigen-based tests and Knott’s examination done on LOE cases referred to LADDL by veterinary practitioners in order to qualify for LOE case reimbursement by pharmaceutical firms. Two candidate dogs (LSU 10 and LSU 13) were identified in separate geographic locations (Figure 2) that fit the four criteria and had sufficient circulating microfilariae for successful experimental infection of laboratory dogs. Dogs were infected using L_3_ raised in *A. aegypti* from circulating microfilariae in blood from candidate dogs [13]. For each pair, one dog was treated with monthly subcutaneous doses of ivermectin of 12 μg/kg (twice the recommended preventive dose for 6 months) and one dog served as an untreated control given a sham subcutaneous dose of propylene glycol at the same monthly interval.

Both treated and untreated dogs infected with the LSU 10 isolate and the LSU 13 isolate developed patent *D. immitis* infections by 218 DPI and 189 DPI, respectively, as evidenced by a positive occult heartworm antigen test and detection of microfilariae by the Knott’s test. Mf counts gradually increased in number of Mf after patency in experimentally infected dogs (Table 1), although microfilaremia in one dog (LSU 10a), initially found in low to moderate numbers, became sporadic with biweekly counts of 2–8 Mf per ml of blood. It is known that up to 25% of naturally infected dogs develop ‘occult’ infections (presence of adult heartworms without circulating Mf). This is thought to occur in some dogs due to development of immunity-mediated removal of circulating Mf [15].

Microfilariae sent to McGill University for genomic studies showed higher genotype frequency of the GG-GG marker [5] loci on P-glycoprotein gene from Mf population from LSU 10 and LSU 13 strain dogs compared to Mf population from LSU 11 and LSU 14 (Table 2). In a separate follow-up study, infective larvae raised from Mf from the treated Group I dog (LSU 10a) were successfully established as a second generation isolate (LSU 10-II), confirming heritability of resistance in the face of a monthly ivermectin challenge dose of 24 μg/kg, 4 times the recommended preventive level for a total of 3 monthly treatments.

We propose that we have successfully isolated two strains from proven cases of resistance that can be used to further study and characterize ML resistance by *D. immitis*.

In a concurrent, related study reported elsewhere, Kaminsky *et al*. [12] reported identification of ML resistant strains using the same animals used for genomic and Mf motility assay studies previously reported [3], providing independent evidence of resistance in three dogs that also originated in LA or AR. Our study and that study are mutually confirmatory and together provide strong evidence of the existence of ML resistant strains of *D. immitis* in the Lower Mississippi Delta.

Resistance to ML and other drugs is well known for nematodes in other host species [18] and the question may be posed as to why it took so long for resistance to emerge as a problem with heartworm ML preventive drugs. The long, essentially annual life cycle of *D. immitis* [19], a large refugia of ‘wild type’ populations in both domestic dogs and wildlife, environmental perturbations of mosquito vectors [20-23], and selection pressure by ‘slow-kill’ adulticide protocols have been proposed as having a role [10,24]. Evidence of resistance to ML has been reported for *Onchocerca volvulus* in long-term preventive ML chemotherapy programs in endemic areas of Africa. It is interesting that it took a similar time frame for *O. volvulus*, approximately 20 years, for documented evidence of ML resistance to emerge in the similarly long life cycle of this filarid species, the cause of human ‘river blindness’ [25].

The role of practicing veterinarians as the first line of defense for detection of adverse drug effects, with FDA, has apparently been effective as a surveillance mechanism in current heartworm control strategies. Moreover, clinical experience gained from practitioners willing to try elective off-label use of drugs and new resistance management strategies promises to lead to new approaches to therapy that can then be proven in controlled published experiments [18,24]. For example, diethylcarbamazine was used for many years for daily use in preventing heartworms but was then replaced by ML drugs after practitioners, kennel owners, and sporting dog groups found that off-label use of recently released ML drugs for livestock, protected against heartworms, intestinal helminthes and other internal parasites with very striking benefits for dogs [26,27]. This was soon followed by controlled pharmaceutical company experimentation leading to FDA approval and marketing in the late 1980’s of current ML drug formulations at the very low dose levels now used for prevention of canine heartworms [26,27].

## Conclusions

These experimental infection studies provide *in vivo* evidence of the existence of ML drug resistance in dogs infected by *D. immitis* L_3_ from suspect field LOE cases in the Lower Mississippi Delta.

The emergence of ML resistance by *D. immitis*, if not controlled in the early stages, threatens to become a widespread problem in the US that limits the effectiveness of current preventive drug treatment methods. There is a need to develop and implement evidence-based resistance management strategies to prevent widespread selection of resistant strains where it has been shown to exist and to suppress potential spread to other areas of the country.

There is current evidence to support the value of potential measures that may be included as part of a resistance management strategy if confirmed by additional research and adopted by the veterinary community. The aim is to suppress spread from dogs harboring resistant *D. immitis* strains and to extend the useful life of currently available drugs:

1) Wider use of the Mf reduction test in veterinary practices to identify suspect resistant cases [8]; 2) Development of laboratory tests to identify resistance markers in suspect cases by microfilariae or adult worms submitted by veterinarians; 3) Confirm that doxycycline/tetracycline therapy can be used to suppress viability of infective larvae that develop in mosquitos from microfilariae of resistant dogs [17] as a way to prevent infection of other dogs where strong evidence of resistance exists (e.g. resistant dogs in the same household or neighborhood or where greatly elevated prevalence of *D. immitis* in mosquitoes is likely after feeding on circulating microfilariae in a resistant dog) [20]; 4) Use of American Heartworm Society recommended adulticide/microfilaricide treatment protocols and avoidance of slow-kill treatment methods in which repeated treatment of infected dogs may potentiate selection of resistance [10]; and 5) Investigate the value of alternative preventative treatment protocols where resistant cases are found, including use of drugs that maintain high, sustained ML blood levels [28-30] or selective return to use of older alternative classes of preventive drugs (ie. daily diethylcarbamazine) [15] pending future development of new alternative drugs.

Additionally, a time-space surveillance system may be warranted to alert the veterinary community on the need for intervention by mapping the location of reported LOE cases and ‘high index of suspicion’ LOE cases that can be confidently attributed to true drug resistance, such as by future genetic marker testing. Regular updates by the FDA on the status of required LOE reports, with other records collected by pharmaceutical industry partners, may serve this purpose. The value of an effective surveillance system may be further enhanced by development of biology-based predictive models on the potential for spread of ML resistance based on climate/environmental suitability determinants, and use of ecological niche models for surveillance using geographic information systems (GIS) risk analysis methods [31-34].

## Abbreviations

LOE: Lack of efficacy
ML: Macrocyclic lactone
Mf: Microfilariae
DPI: Days post-infection
FDA: Food and Drug Administration
LADDL: Louisiana Animal Disease Diagnostic Laboratory
LVMA: Louisiana Veterinary Medical Association
GIS: Geographic information systems
ᅟ: LSU 10, LSU 11, LSU 13, LSU 14 strain designation of source/isolate
